# Ethnographic study of ICT-supported collaborative work routines in general practice

**DOI:** 10.1186/1472-6963-10-348

**Published:** 2010-12-29

**Authors:** Deborah Swinglehurst, Trisha Greenhalgh, Michelle Myall, Jill Russell

**Affiliations:** 1 Healthcare Innovation and Policy Unit, Centre for Health Sciences, Barts and The London School of Medicine and Dentistry, London E1 2AT, UK; 2 Senior Researcher, WRC Research, Ground Floor East, 33-41 Dallington Street, London EC1V 0BB, UK

## Abstract

**Background:**

Health informatics research has traditionally been dominated by experimental and quasi-experimental designs. An emerging area of study in organisational sociology is routinisation (how collaborative work practices become business-as-usual). There is growing interest in the use of ethnography and other in-depth qualitative approaches to explore how collaborative work routines are enacted and develop over time, and how electronic patient records (EPRs) are used to support collaborative work practices within organisations.

**Methods/design:**

Following Feldman and Pentland, we will use 'the organisational routine' as our unit of analysis. In a sample of four UK general practices, we will collect narratives, ethnographic observations, multi-modal (video and screen capture) data, documents and other artefacts, and analyse these to map and compare the different understandings and enactments of three common routines (repeat prescribing, coding and summarising, and chronic disease surveillance) which span clinical and administrative spaces and which, though 'mundane', have an important bearing on quality and safety of care. In a detailed qualitative analysis informed by sociological theory, we aim to generate insights about how complex collaborative work is achieved through the process of routinisation in healthcare organisations.

**Discussion:**

Our study offers the potential not only to identify potential quality failures (poor performance, errors, failures of coordination) in collaborative work routines but also to reveal the hidden work and workarounds by front-line staff which bridge the model-reality gap in EPR technologies and via which "automated" safety features have an impact in practice.

## Background

### The need for qualitative studies of organisational routines

The study of innovation and change in healthcare organisations is, arguably, under-theorised and in need of methodological enrichment. Research has focused predominantly on experimental, quantitative and (often) behaviourist study designs oriented to developing interventions, testing hypotheses and measuring the relationship between inputs (e.g. training), processes (e.g. following a guideline) and outcomes (e.g. morbidity). These empirically-driven ('positivist') approaches resonate strongly with medicine's methodological hierarchy of evidence in which the controlled experiment counts highly [1].

Important though such research is, there is also a need for in-depth qualitative research ('interpretivist' approaches) oriented to developing theories and explanations of *how *innovation and change happens - and in particular, how new ideas, practices and collective behaviours become *routinised *as business-as-usual. The need to research routinisation is particularly pressing given that as healthcare becomes ever more complex and multi-professional, the limited penetration of potentially effective innovations as well as a high and rising proportion of quality and safety failures are all attributed to poor communication and coordination between groups and teams [2,3].

Our study seeks to contribute to a body of knowledge which lies at the interface between health services research and organisational sociology. Our 2004 systematic review on diffusion of innovations in healthcare organisations identified numerous studies of individual adoption but highlighted a dearth of research on the process by which innovations become routinised at organisational level [4]. A later update of that systematic review identified an emerging literature on routinisation [5]; we explored its implications for healthcare in a further paper [6]. A recent systematic review on implementation of electronic patient records (EPRs) in organisations revealed a preponderance of experimental and quasi-experimental studies and a much smaller qualitative literature describing the social processes and contextual influences on EPR adoption and use [7].

### Tensions in organisational research

Scholars in organisational sociology tend to frame the study of innovation and change not in terms of interventions and outcomes but as the playing-out of tensions: between the general unwritten rules and forces which make up society ('social structures') and individual behaviour ('agency') [8]; between collective knowledge and individual knowledge [9]; and between continuity and change [10]. Sociological studies of information and communication technologies (ICTs) in organisations add a fourth tension: between standardisation and contingency [7,11-13]. Health informatics scholars have generally shown more interest in promoting standardisation (e.g. developing common codes and interoperability standards) than exploring contingency (e.g. a team's commitment to a stand-alone legacy system whose limitations and the workarounds for overcoming them are part of local business-as-usual), though in a companion paper we review some ethnographic studies on local EPR systems (Greenhalgh T, Swinglehurst D: Studying technology use as social practice: the untapped potential of ethnography, submitted). More generally, the researcher's challenge is usually seen as rising above ephemeral, situated detail in the search for abstracted, generalisable truths.

Harold Garfinkel, the father of ethnomethodology, bucked this trend, arguing that the organisational researcher's main focus should be the *non-generalisable *particularities of small-scale social situations [14]. He argued that each utterance, written comment or action occurs in a micro-sequence that takes detailed and tacit account of the utterances, comments or actions preceding it, and proposed that it is these subtle contingencies of work, not the abstract routines and patterns an observer might see 'sedimenting' from them, which are of greatest interest [15]. Both perspectives, of course, are important.

### Organisational routines

To routinise an innovation is to embed it into routines. Organisational routines have been defined as *"repetitive recognisable patterns of interdependent actions by multiple actors" *[16]. Routines (which include such things as ward rounds, meetings, surgical operations and making telephone bookings) are the way organisational life is patterned [6]. A routine conveys complex, tacit knowledge and also serves to coordinate and control. Early theoretical work on organisational routines emphasised their abstracted qualities, especially the common characteristics of a particular routine across different enactments of it, and the contribution of routines to organisational stability [17]. But Feldman and Pentland drew attention to the situated (local, one-off) nature of every routine and its critical dependence on human actors who embody the routine, embrace it or resist it, and put greater or lesser creative effort into improving it and/or shaping it to the particularities of the here-and-now [18].

**Figure 1:**
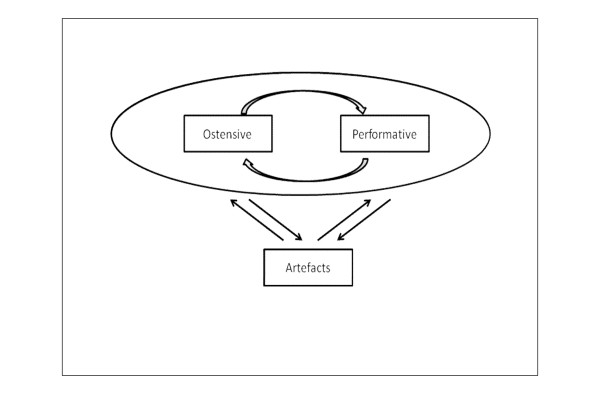
**Key aspects of the organisational routine.** Reproduced from original article with permission of Oxford University Press [19].

The production and reproduction of organisational routines by human actors is a specific example of the structure-agency tension described by Giddens in structuration theory [8]. Pentland and Feldman suggest a model of the routine which incorporates both ostensive (the abstract understanding or 'script' of the routine-in-general which actors might describe if asked) and performative (what particular people actually do in particular situations, paying attention to the actions of particular others and with a particular goal in mind) [19]. Artefacts (such as standard operating procedures, guidelines, protocols and so on) may codify the intended steps in a routine but should not be equated with what actually gets done (see Figure 1).

There is no one 'true' ostensive version of a particular organisational routine. Rather, there are multiple, overlapping typifications and understandings which guide and account for particular performances of it. The situated nature of the performative routine is important [20]. Individual work is an effortful accomplishment in which participants use their discretion as they select from a repertoire of possibilities, or 'organisational grammars' [21]. Organisational members learn from previous iterations of a routine and mindfully seek to make sense of actions-in-context by drawing on their understanding of the wider organisation [16,22]. This emphasis on human creativity and effort contrasts with earlier notions that routines are 'mindless' (i.e. repetitive and semi-automated) [23].

There is an ongoing tension between any individual's contribution to a routine and the routine as a whole. Interpretivist research has long emphasised the principle of the hermeneutic circle - that is, the need to analyse the parts in detail while maintaining awareness of the whole and relate micro-level findings to this wider picture [24]; this has been applied to ICT research [25] and to the study of routines [17]. The organisational ethnographer must shift between studying the work of individuals within a particular routine, the overall routine, and the wider organisational context, with reflexive awareness of the dynamic interplay between parts and wholes.

The development (or attrition) of routines over time reflects the more general tension between continuity and change in organisational life [10]. The routine (noun) is linked to efforts to routinise (verb) - a key step in introducing new ideas and service models [6]. But even once routinised, an innovation must adapt to a changing context and to a continuous stream of other innovations and changes. Routines are differently reproduced every time they are enacted, because different people bring different prior knowledge, expectations, priorities, assumptions, personalities and skills to their work and are enabled and constrained by different local influences, both social and technical. Herein lies the scope for organisations to learn and for particular routines to move flexibly with the times [18].

### Researching routines dynamically

Analysing the divergence between ostensive and performative aspects of routines and the artefacts through which members attempt to codify and capture these can reveal rich meanings in aspects of organisational life (data entry, telephone calls, administrative notes) which were previously considered mundane, uniform and offering little in the way of research insights [19]. Divergence between artefacts and ostensive routines (often overlooked since the artefact is assumed to *be *the routine), for example, may highlight failures of sensemaking, conflicts between management and staff, or conflicts between the organisation and a wider public. For example, a 'health and safety' poster may be displayed within a reception area as a legal requirement but have little or no impact on individual or organisational routines relating to health and safety. Divergence between artefacts and performative routines may reveal organisational power struggles - most commonly when management introduce formal protocols in an effort to control behaviour, but these *representations *of recurrent action patterns fail to give rise to *actual *recurrent action patterns [26].

### The electronic patient record as 'actor'

Health informatics research conventionally portrays the EPR in terms of its essential, intrinsic properties as a 'container' for data about the patient (and perhaps, as a medicolegal record or source of secondary data). But research in fields such as sociology, actor-network theory and computer-supported cooperative work views the EPR in more dynamic terms - as an active player in an ever-changing (and often unstable) network of people and technologies [7]. This is not to suggest that the EPR has human-like agency. Rather, the focus of this alternative literature is to consider the EPR in *relational *terms - that is, in terms of what it *becomes *when part of a particular socio-technical network [27]. This dynamic view of the EPR links elegantly to the literature on organisational routines described above and offers exciting possibilities for studying change in healthcare organisations through a novel, socio-technically informed analytic lens.

The aims of this study are (at an empirical level) to explore the use of EPRs in collaborative work routines in general practice and (at a more abstract level) to develop theory and method which will inform a wider programme of qualitative research into ICT-supported collaborative work, innovation and change in healthcare organisations. At a theoretical level, we are interested in exploring how key organisational tensions (collective-individual, continuity-change, standardisation-contingency) play out over time and across settings via enactment of routines.

## Methods/design

### Research question

How are collaborative work routines enacted, and how do they develop and change over time in healthcare organisations? What is the role of information and communication technologies (specifically, the electronic patient record) in shaping, constraining and perpetuating this process?

### Study design and setting

Multi-centre case study in four UK general practices.

### Study objectives

1. To conduct detailed ethnographic observation of collaborative work involving the EPR in participating organisations over a period of time.

2. To map how selected collaborative routines are codified (artefactual or proxy routine), understood (ostensive routine) and enacted (performative routine) by staff in those organisations.

3. To compare and contrast different versions of the routines within each organisation with a view to illuminating how key organisational tensions play out dynamically over time.

4. To compare findings across cases and through time with a view to making theoretically-informed generalisations about the routinisation process.

### Intended outputs

We hope to generate four main outputs:

1. Four detailed case studies describing EPR-supported collaborative work routines in general practice.

2. A transferable methodology for the detailed qualitative study of ICT-supported collaborative work in healthcare organisations.

3. Theoretical insights into how ICT-supported routines develop and evolve (or not) in healthcare organisations.

4. Hypotheses for further research on how to introduce and routinise ICT innovations intended to improve quality and/or safety of care.

### Management and governance

Research ethics approval has been granted by Thames Valley Multi-centre Research Ethics Committee (06/MRE12/81). An external steering group with a lay chair has been established and meets four-monthly throughout the 3-year research period. Core team meetings occur monthly.

### Selection of organisational cases

The selection criteria for the sample of four general practices are [a] *opportunity to learn *and [b] representativeness. Stake's approach to organisational case study views this as a fundamentally interpretive process in which generalisations are made by theoretical, not statistical, abstraction (i.e. a rigorous case study analysis is one in which events and actions are linked via a plausible and richly-theorised account) [28]. With this in mind, opportunity to generate learning is identified via features such as interest in the study, willingness of staff at all levels to participate in the research process, plausibility of planned data collection methods (e.g. adequate physical space), and evidence of the organisation's engagement with previous comparable studies. Practices meeting these criteria will be selected for diversity in terms of size, geographical setting, demographics of population served and sophistication of in-house ICT systems.

### Selection of routines to be studied

Contemporary general practice in the UK is characterised by low incidence of major emergencies; high level of computerisation oriented to both primary uses of data (patient care) and secondary uses (audit, research, surveillance, implementing quality incentives) [29]; an increasing focus on chronic disease management and risk assessment (which depend on registration, recall and regular review) [30,31]; a well-demarcated division of labour, with patient care tending to be divided into tasks and delegated to the cheapest individual able to complete each task [32]; and a growing patient safety agenda, especially in relation to medicines management (i.e. prevention of drug-related errors and adverse reactions) [33].

Because of the above characteristics, we are particularly interested in studying routines which [a] are oriented to 'everyday' general practice rather than emergencies; [b] span both clinical and administrative work; [c] involve both primary and secondary uses of the electronic record; [d] require collaboration between staff both synchronously and asynchronously in time and space; and [e] address the quality and/or safety agenda. We have chosen three such routines for further study, namely:

1. Issuing repeat prescriptions

2. Summarising and coding (e.g. of outpatient and discharge letters)

3. Surveillance of chronic disease

Identifying and exploring routines will not be an end in itself. Indeed, the detailed tasks, processes and interactions for (say) repeat prescribing are of limited intrinsic interest. They are, however, a way of opening up to scrutiny the interaction between the EPR, its users, the general practice organisation and wider influences (e.g. policy directives). By synthesising and comparing routines across a sample of practices, we aim to produce generic insights into the EPR as a technology-in-use [34] - and at a more abstract level, insights into how socio-technical micro-systems contribute to both perpetuating and changing collaborative routines in healthcare organisations.

### Data sources and collection methods

**Figure 2:**
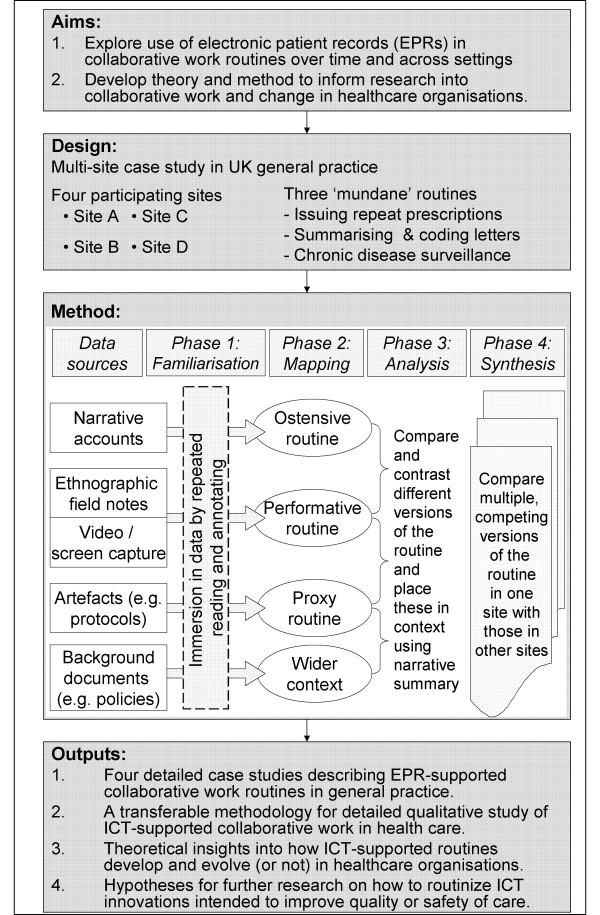
**Study protocol**.

Figure 2 shows the key data sources for this study. These comprise:

• *Narrative accounts *of front-line staff in which they describe their work. Narrative accounts will be collected naturalistically (i.e. in the real environment of work rather than a formal interview situation) since it is well documented that people describe a work process better when they are actually doing it or close to someone who is doing it [35]. Between 15 and 30 such accounts (5-10 per routine) will be collected in each organisation. As part of this dataset, we will ask staff to "talk us through" particular tasks and procedures and show us how (if at all) they draw on formal artefacts such as templates or protocols (or informal ones such as handwritten notes) when undertaking these. We will also explore their understanding of other members' contributions to the same routine.

• *Ethnographic observation *of staff undertaking the routine. Experienced researchers will sit in on both clinical and administrative work and make notes on the actions and talk of staff engaged in the real-time enactment of target routines. We anticipate spending between 100 and 150 hours in each organisation. Studying repeat prescribing, for example, will include observing administrative staff sorting requests, printing prescriptions, 'querying' requests and processing the signed prescriptions from clinicians, and also observing clinicians signing the prescriptions and responding to queries; messages exchanged between staff on post-it notes and via internal email will be part of this dataset.

• *Video and screen capture*. In order to supplement direct ethnographic observation of clinical consultations in relation to chronic disease surveillance, we will follow Jewitt's methodology for collecting multi-modal data [36]. Subject to consent of both parties, we will use an unobtrusive video camera positioned so as to view the faces of clinician and patient. We will also use screen capture software to record what is entered in real time onto the EPR. We will aim to collect data on 10-15 such consultations in each practice (40-60 in total).

• *Artefacts*. We will collect from the general practices any documentation describing the target routines or parts of routines to staff, patients or other parties. In relation to repeat prescribing, for example, these may include: staff protocols, training or induction materials, internal memos, algorithms, and relevant sections of the practice leaflet and website.

• *Background documents*. We will also collect documentation relevant to the wider context such as practice annual reports, and relevant local and national guidelines and policies (e.g. on medicines management or chronic disease surveillance).

### Data mapping and analysis

In an initial familiarisation phase (see Figure 2), we will read, re-read and annotate field notes, transcripts and other texts and also view video data repeatedly to achieve immersion in the data [37]. This will feed into a mapping phase, in which we will identify and refine a picture of [a] the ostensive routine (i.e. the sometimes conflicting narrative accounts and typifications which members give when asked to describe what is done) including, where relevant, the use of space and time as structuring devices; [b] the performative routine as directly observed, paying close attention to practices, puzzles faced, dilemmas encountered, people involved and language used; and [c] the proxy routine as depicted in artefacts such as protocols, guidelines, templates, patient leaflets and so on. We will avoid trying to 'isolate out' the EPR but will study this inasmuch as it is integral to the routine we are mapping.

In the analysis phase, we will compare ostensive, performative and proxy routines, considering interfaces and divergence between these and using narrative to draw the analytic threads together and interpret the multiple, competing versions of the routine in context. In this way, we will generate preliminary explanations of how collaborative work occurs and how the target routines are perpetuated and shaped by both human agency and the functionality of the EPR. We will use narrative accounts, ethnographic notes and video and screen capture data to "zoom in" on the micro-detail of small-scale incidents and interactions, and also use our wider data sources within and beyond the organisation to "zoom out" and consider external influences, thus placing the routine in wider context (Swinglehurst D, Roberts C, Greenhalgh T: Opening up the "black box" of the electronic patient record: a linguistic ethnographic study in general practice, submitted). Finally, in a synthesis phase we will compare how routines vary both over time in a single organisation and across the different general practices in our sample.

## Discussion

We have piloted the data collection methods in two general practices and found them feasible and acceptable to staff. Focusing on organisational routines rather than individual performance or outputs has helped significantly in gaining access and establishing productive research relationships, especially amongst non-clinical staff. Participants understand that detailed observation of their work is essential for us to build a picture of the whole routine, and appear very willing to talk us through work practices, giving us access to the ostensive routine through naturally occurring talk [38]. We have confirmed Barley and Kunda's finding that knowledge of parts of routines held by individual actors is largely tacit and hard to articulate [35]. One administrator, for example, commented: "*I have been doing this so long, my fingers go faster than my brain. I don't really know what I am doing any more*".

Our chosen research focus (the collaborative work which the EPR supports) was driven mainly by our theoretical position described above, and we rejected the more narrow and static focus on the technology itself preferred by some previous researchers. It is, however, worth noting that when piloting our methods, we have been struck by the impossibility of isolating out the EPR or its 'impact' when making ethnographic field notes. Staff roles cannot be described separately from their engagement with the EPR, and conversely, the EPR cannot be meaningfully described without constant reference to who is using it. For example, receptionists in one practice talked of being "on the computer" - which (in that setting) meant issuing repeat prescriptions.

Despite pertaining to what appear on the surface to be relatively simple tasks and processes, the routines we are seeking to explore do not 'fall out of the data'. Previous researchers have presented a somewhat reified picture of organisational routines as readily-discernible patterns of action and interaction which are 'out there' in the organisation, ready to be researched [23]. In reality, as the preliminary data fragments in Table 1 show, the ostensive, performative and proxy routines for scanning and coding incoming letters are social constructions which are differently perceived by different organisational members. This messiness is not unsurprising but will require careful attention to the 'immersion' and 'mapping' phases in Figure 2.

**Table 1:** Data fragments illustrating ostensive, performative and proxy routine for scanning and coding incoming letters

TYPE OF ROUTINE	EXAMPLE
**Ostensive routine**	**From researcher's summary based on narratives of practice staff** *The 'old' system involves the doctors highlighting in pen on the letter the things they want READ coded (ring round) or added as free text (scored through with highlighter pen). With DOCMAN [a recent add-on to the EMIS electronic record software], a letter is received by the practice, stamped with a date stamp which also has other things on the stamp (Problem Title; Date; Active; Past; Minor; GP init; sum; s/c (meaning scanned)). X [receptionist] said that the person scanning the letter initials it. The other fields on this stamp are essentially not used. The letter is then scanned and added to DOCMAN. It is then sent electronically through DOCMAN for viewing/highlighting by the GP*.

**Performative routine**	**From field notes of direct observation of the routine** *"I asked Z [secretary] if it was OK if I watched her sorting post next door and she was fine about that. Everything was date stamped. She explained that the stamp indicated that the letters had been scanned (but they hadn't - they had just come out of the envelopes). She explained that if a GP sees a letter without a date stamp on it they know that it is not scanned so it needs to be put back in the sec's tray. She said that X [fellow secretary] didn't stamp until after scanning - but that they both do things slightly differently. She had made a separate pile of letters which were printed on both sides and took those to the photocopier to photocopy the 'back' side of these letters which made it much easier to put them through the scanner. (again she pointed out X doesn't do this)."*

**Proxy routine**	**As depicted in formal protocol** Coding - a how to guide: All written correspondence and test results that the Practice receives is scanned into the records of the relevant patient. Certain types of correspondence are also read coded to enable the information to be found by running searches. Items that need to be coded are detailed below. Read codes These are unique codes made up of a combination of up to 4 letters and numbers. There are read codes relating to almost everything - being sucked into the jet of a space craft, being bitten by a crocodile whilst at home and drowning accidentally (as though people often drown on purpose) whilst pearl diving. Logging information under its specific read code means that it can be easily retrieved - eg a search for code 621 would bring up all women who are currently pregnant. In this way we can keep on top of all our patients with particular conditions.

We have deliberately chosen to study routines which span what Goffman calls 'front-stage' work (e.g. carried out by clinicians in their consulting rooms) and the 'back-stage' activities which support and augment this work [39]. For example, in a pilot observation, an administrator referred to "doing the baby clinic". What she was actually referring to was her own specific role of entering vaccination batch numbers into a computer template in the electronic record, but this was part of a wider organisational routine known simply as "baby clinic" which also, at different times and in different spaces, included clinical staff (and, at some point, babies). Drawing out the routine as a whole across both clinical and administrative space, rather than simply focusing on one person's role in it, will allow us to depict how the EPR is not merely a 'container' onto which doctors and nurses enter data but a 'player' in complex collaborative working practices right across the organisation.

We have found that material artefacts - such as practice protocols, electronic templates for chronic disease management, and patient information (e.g. a practice leaflet about a new online appointment-booking system) - are readily gathered. More subtle artefacts which reflect how designers expect a routine to be enacted include the layout of a room (such as whether clinicians consult across a desk or obliquely so that the patient can see the computer screen) or seating arrangements (indeed, some routines seem to be defined as much by *where *they are undertaken as by *what *is being done by the staff member). As the data fragments in Box 1 show, artefacts sometimes reveal an expectation that a particular task (such as scanning and attaching documents to the electronic record) is uniform and mundane when in reality it is (to a greater or lesser extent) unpredictable and demanding.

As Table 1 shows, the subtle mismatches between the proxy routine depicted in the formal protocol (artefact), the mental models which staff carry in their heads (ostensive routine) and what actually gets done (performative routine) illustrate a fertile area for quality and safety research. However, it would be wrong to assume that the 'gold standard' is captured in the artefact and that any deviation from this should be classified as a potential threat to quality or safety, since as Hartswood and Procter have previously shown in relation to administrative work in breast cancer screening [40]) staff may develop workarounds and other 'protocol deviations' as deliberate or unconscious measures to *increase *quality and safety. For example, whilst the formal protocol for repeat prescribing is that a doctor checks and signs each prescription, receptionists may observe that in reality, doctors do not check each medication before signing, and hence add an informal safety measure (e.g. a post-it note asking "OK to give?").

We are particularly keen to explore how the informal workarounds and articulations introduced by front-line staff to improve quality or safety interface with the EPR's automated prompts and inbuilt design features. Pop-up decision support prompts, for example, may be 're-localised' by reception or administrative staff [41], who may (sometimes but perhaps not always) send an informal message to a clinician to say "computer is asking about...". These complex and subtle interactions between the EPR's standard prompts and situated human judgements will form a major focus of the analysis.

In summary, we have described an innovative study design and methodology for studying the micro-detail of EPR-supported collaborative work in general practice. In a sample of four UK general practices, we will collect narratives, ethnographic observations, multi-modal data, documents and other artefacts, and analyse these to map and compare the different understandings and enactments of selected organisational routines which span clinical and administrative spaces and which have an important bearing on quality and safety of care. In a detailed analysis informed by sociological theory, we aim to generate insights about how ICT-supported collaborative work is achieved in healthcare organisations. Our study offers the opportunity not only to identify potential quality failures (poor performance or error) but also to reveal the hidden work (and workarounds) by front-line administrative and clinical staff via which "automated" safety features of technology are adopted and have an impact in practice.

## Competing interests

The authors declare that they have no competing interests.

## Authors' contributions

TG conceptualised the study with input from DS and JR. DS and MM developed and piloted the data collection methods, helped by TG and JR. DS and MM collected the data. DS and TG wrote the paper. All authors read and approved the final manuscript.

## Pre-publication history

The pre-publication history for this paper can be accessed here:

http://www.biomedcentral.com/1472-6963/10/348/prepub
